# A unified computational model of the genetic regulatory networks underlying synaptic, intrinsic and homeostatic plasticity

**DOI:** 10.1186/1471-2202-12-S1-P161

**Published:** 2011-07-18

**Authors:** Daniel Bush, Yaochu Jin

**Affiliations:** 1 Department of Computing, University of Surrey, Guildford, Surrey, GU7 2XH, UK

## 

It is well established that the phenomena of synaptic, intrinsic and homeostatic plasticity are mediated – at least in part – by a multitude of activity-dependent gene transcription and translation processes [1-3]. Various isolated aspects of the complex genetic regulatory network (GRN) underlying these interconnected plasticity mechanisms have been examined previously in detailed computational models [4,5]. However, no study has yet taken an integrated, systems biology approach to examining the emergent dynamics of these interacting elements over longer timescales. Here, we present theoretical descriptions and kinetic models of the principle mechanisms responsible for synaptic and neuronal plasticity within a single simulated Hodgkin-Huxley neuron. We describe how intracellular Calcium dynamics and neural activity mediate synaptic tagging and capture (STC), bistable CaMKII auto-phosphorylation, nuclear CREB activation via multiple converging secondary messenger pathways, and the activity-dependent accumulation of immediate early genes (IEGs) controlling homeostatic plasticity. We then demonstrate that this unified model allows a wide range of experimental plasticity data to be replicated. Furthermore, we describe how this model can be used to examine the cell-wide and synapse-specific effects of various activity regimes and putative pharmacological manipulations on neural processing over short and long timescales. These include an examination of the interaction between intrinsic and synaptic plasticity, each dictated by the level of activated CREB; and the differences in functionality generated by STC under regimes of reduced protein synthesis [2,6]. Finally, we discuss how these processes might contribute to maintaining an appropriate regime for transient dynamics in putative cell assemblies within contemporary neural network models of cognitive processing [7,8].
